# KRP-DS: A Knowledge Graph-Based Dialogue System with Inference-Aided Prediction

**DOI:** 10.3390/s23156805

**Published:** 2023-07-30

**Authors:** Qiang He, Shuobo Xu, Zhenfang Zhu, Peng Wang, Kefeng Li, Quanfeng Zheng, Yanshun Li

**Affiliations:** School of Information Science and Electrical Engineering, Shandong Jiaotong University, Jinan 250357, China; 21208022@stu.sdjtu.edu.cn (Q.H.); zhuzf@sdjtu.edu.cn (Z.Z.); 205049@sdjtu.edu.cn (P.W.); 205073@sdjtu.edu.cn (K.L.); 21208007@stu.sdjtu.edu.cn (Q.Z.); 21208021@stu.sdjtu.edu.cn (Y.L.)

**Keywords:** intelligent dialogue system, chat bots, knowledge-grounded dialogue, knowledge graph

## Abstract

With the popularity of ChatGPT, there has been increasing attention towards dialogue systems. Researchers are dedicated to designing a knowledgeable model that can engage in conversations like humans. Traditional seq2seq dialogue models often suffer from limited performance and the issue of generating safe responses. In recent years, large-scale pretrained language models have demonstrated their powerful capabilities across various domains. Many studies have leveraged these pretrained models for dialogue tasks to address concerns such as safe response generation. Pretrained models can enhance responses by carrying certain knowledge information after being pre-trained on large-scale data. However, when specific knowledge is required in a particular domain, the model may still generate bland or inappropriate responses, and the interpretability of such models is poor. Therefore, in this paper, we propose the KRP-DS model. We design a knowledge module that incorporates a knowledge graph as external knowledge in the dialogue system. The module utilizes contextual information for path reasoning and guides knowledge prediction. Finally, the predicted knowledge is used to enhance response generation. Experimental results show that our proposed model can effectively improve the quality and diversity of responses while having better interpretability, and outperforms baseline models in both automatic and human evaluations.

## 1. Introduction

In recent years, conversational systems have experienced rapid development and conversational quality has been continuously improved thanks to the rapid growth of deep learning and massive conversational data on the Internet. They have demonstrated significant potential for development and commercial value across various fields, drawing extensive attention from both the industry and academic communities [1]. Our work primarily focuses on open-domain generative conversational systems, which have the advantage of providing more flexible and diverse responses, unconstrained by tasks and domains.

Open-domain generative models for dialogue often face criticism for generating dull and safe responses, such as “I don’t know” or “Okay.” These responses contribute to meaningless and tedious conversations [2]. To address this issue, some research efforts have applied large-scale pre-trained language models like GPT2 [3] to open-domain dialogue generation tasks, such as CDial-GPT2 [4] and DialoGPT [5]. These transformer models trained on extensive datasets can capture long-term contextual dependencies in dialogue data and generate diverse and fluent responses [6]. However, when dialogues require specific and domain-specific knowledge, these models still tend to produce bland or inappropriate replies. As shown in Table 1, for ordinary responses and responses enhanced with external knowledge, we present the responses generated by two different dialogue models: one composed solely of the pre-trained language model BART, and our KRP-DS model.

Under the same input context, BART often generates safe responses. BART, as an encoder–decoder framework, heavily relies on the context information and parameterized memory during response generation, resulting in limited knowledge acquisition. In contrast, our KRP-DS model leverages external knowledge graphs to generate high-quality responses that incorporate knowledge. Thus, it is evident that incorporating external knowledge is essential for generating appropriate and informative responses. To address this, several approaches have employed structured knowledge graphs (KGs) as external knowledge sources [7]. Knowledge graphs (KGs) represent external knowledge in a structured form, consisting of entities and relationships [8]. Since the selected knowledge determines the model’s final response, it is crucial to utilize the context effectively and predict appropriate knowledge. Numerous efforts have focused on these aspects. Reference [9] employs the knowledge representation learning algorithm TransE to obtain embedded representations of knowledge triplets in the knowledge graph, and enhances the understanding of context and improves response generation through static and dynamic graph attention mechanisms. Reference [10] introduces a key-value storage module in the model to leverage knowledge information, treating the context as a query to retrieve relevant knowledge during the decoding step, where entities and relationships are represented using average word embeddings. Reference [11] constructs an MHKT-Path by leveraging all relations and triplets in the context. It can be seen as a subgraph of the external knowledge graph, incorporating both implicit knowledge from the context and structural information from the knowledge graph, which can better guide the model in knowledge prediction. Although optimizing knowledge prediction in this way enables the selection of more suitable knowledge for generating informative responses, the method of acquiring knowledge in this manner has limited interpretability. Therefore, some works [12,13,14,15] employ knowledge graph path traversal to represent the knowledge reasoning process, enhancing the transparency and interpretability of dialogue systems. However, these methods still have some limitations. Some methods focus solely on knowledge entities while neglecting the importance of relationships, others only predict inference paths without generating responses, and some employ relatively simple RNNs, failing to achieve optimal performance.

In this paper, we propose the KRP-DS model, which leverages large-scale pretrained models as the backbone to acquire strong foundational dialogue capabilities and knowledge. We designed a knowledge module that utilizes a knowledge graph as external knowledge and employs the TransD algorithm [16] to obtain knowledge graph embeddings. TransD, compared to other knowledge representation algorithms such as TransE and TransR, offers lower complexity and greater flexibility, enabling better knowledge representation. The module also employs multi-hop reasoning based on the relationship sequence to guide knowledge prediction, achieving a balance between performance and model interpretability. Finally, we integrate the context and predicted knowledge triplets to generate informative and fluent responses.

The main contributions of this paper are as follows:

- We propose a knowledge graph-based open-domain dialogue system model called the KRP-DS model.
- We have designed a knowledge module that utilizes a knowledge graph represented by the TransD algorithm [16] as external knowledge. The module employs knowledge graph reasoning to guide the prediction of knowledge triplets, resulting in interpretable reasoning paths and predicted knowledge triplets. This approach enhances the quality and interpretability of the generated responses.
- Experimental validation on the KdConv dataset confirms the effectiveness of our model, demonstrating superior performance over the baseline models in both automatic and human evaluations.

## 2. Related Work

Earlier works on dialogue systems [17,18] typically employed seq2seq models. References [19,20] extended and improved the encoder and decoder to enhance response diversity. With the advent of the pre-training era, the use of various pre-trained language models based on the Transformer [21] have become mainstream in dialogue systems. DialoGPT [5] pretrains the GPT-2 [3] model on a large-scale conversational corpus and applies it to dialogue generation. Meena [22] adopts the Evolved Transformer [23] architecture, utilizes more dialogue data, and employs a larger model, achieving better performance. PLATO [24] addresses the one-to-many problem in dialogue generation by employing discrete latent variables. GODEL [25] introduced a novel approach based on grounded pre-training. However, since these models can only implicitly learn commonsense knowledge from dialogue corpora, they may struggle to apply this knowledge to other dialogue scenarios.

The introduction of external knowledge has proven to be effective in improving the quality and appeal of responses, and extensive research has been conducted on how to leverage external knowledge to guide better response generation. One representative model is the memory network [26]. Knowledge-based dialogue systems store external knowledge in memory networks and retrieve relevant knowledge based on the dialogue to assist in generating responses during the generation process [27]. Some studies [28,29] utilize posterior probability distribution to assist in knowledge selection for choosing more appropriate knowledge, thereby improving the quality of responses. Reference [2] applied large-scale pretrained language models to knowledge-driven dialogue generation and proposed a knowledge selection module. Additionally, in the training phase, simultaneous optimization of knowledge selection and dialogue generation was conducted. Reference [9] attempted to incorporate large-scale KGs into end-to-end dialogue systems. Reference [11] employed MHKT-Path to capture the implicit knowledge in the context and the transformation patterns of knowledge graph relations, thus further improving knowledge prediction and response generation. These systems may retrieve suitable knowledge from the KG, but they do not provide interpretability.

Therefore, some works [12,13,14,15] explicitly represent the reasoning process as path traversal on the knowledge graph. These methods further enhance the transparency and interpretability of conversational agents. However, some of them overlook the importance of relationship information: some only predict reasoning paths without generating responses, some solely focus on the last utterance to select knowledge, and some utilize traditional RNNs as the backbone without leveraging the advantages of pre-trained language models.

In this paper, our model incorporates interpretable knowledge prediction, acknowledges the role of entity relationships, and fully utilizes the entire context information rather than just the last utterance to guide knowledge selection. We employ pretrained language models to achieve better basic conversational performance.

## 3. Model

### 3.1. Task Formulation and Model Overview

In our task, each training sample consists of a set of dialogue contexts *C*, the corresponding ground truth response *Y*, and the associated knowledge triplets *K* and knowledge text *T*, where $C=\left\{u_{1},u_{2},\ldots ,u_{n-1}\right\}$ represents the dialogue history of *n* − 1 rounds, and the *n*th round of dialogue serves as the true response *Y*. Each round of dialogue is annotated with knowledge graph triplets and knowledge text. $K=\left\{kg_{1},kg_{2},\ldots ,kg_{i}\right\}\left(i\geq 0\right)$ represents the set of knowledge triplets relevant to the dialogue, where each $kg=\left(h,r,t\right)$ represents a knowledge triplet, with *h* denoting the head entity, *r* denoting the relation, and *t* denoting the tail entity. $T=\left\{kt_{1},kt_{2}\ldots ,kt_{i}\right\}\left(i\geq 0\right)$ represents a collection of knowledge texts related to the conversation, where each *kt* is a paragraph of knowledge description text. The goal of our model is to generate high-quality responses based on the dialogue context, relevant knowledge triplets, and knowledge text.

**Figure 1:**
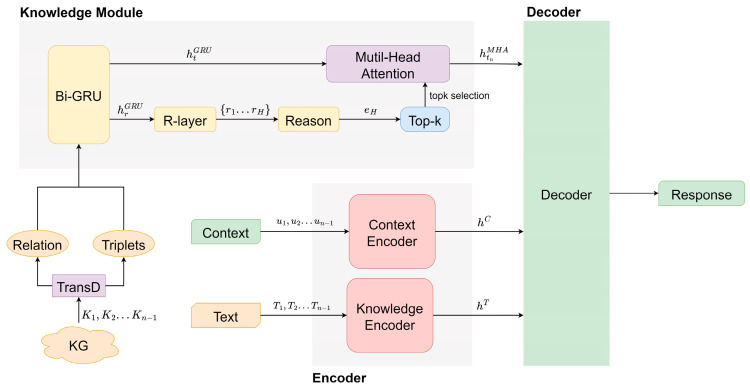
An overview of the proposed model KRP-DS. The encoder module encodes both the context and the knowledge texts separately. The knowledge module predicts the knowledge required for generating the response based on the knowledge triplets appearing in the context and inferred entities. The decoder module takes the concatenated input of the context, text, and predicted triplets, and generates the response as the output.

The structure of the KRP-DS model is illustrated in Figure 1, consisting of three components: an encoder (composed of a context encoder and a knowledge text encoder), a knowledge prediction module that integrates reasoning, and a knowledge-enhanced decoder. We will now provide detailed explanations of each module.

### 3.2. Encoder

In this paper, we utilize the encoder of the pretrained model BART [30] as our contextual and knowledge text encoder. The contextual encoder encodes the dialogue context for each round and obtains semantic representations. First, special tokens [CLS] and [SEP] are inserted before and after the dialogue history $C=\left\{u_{1},u_{2},\ldots ,u_{n-1}\right\}$ to mark the beginning and end of the conversation. [SEP] is also used to separate each utterance and indicate the boundary between them. Then, the input text is tokenized using a tokenizer, splitting it into individual tokens. These tokens are then mapped to corresponding integer indices. Each token corresponds to a unique index in the vocabulary. Then, the input is passed through the encoder, resulting in the final contextual representation:

(1) $$h^{C}=Encoder_{bart}\left(C\right)$$

where *C* represents the dialogue context, Encoder refers to the encoder implemented by BART, and $h^{C}$ represents the semantic representation of all tokens in the context. The knowledge text encoder follows a similar approach. For each dialogue round, the corresponding set of knowledge texts $T=\left\{kt_{1},kt_{2}\ldots ,kt_{i}\right\}\left(i\geq 0\right)$ is separated by [SEP] to tokenize and encode each sentence. The semantic representation of is obtained as follows:

(2) $$h_{}^{T}=Encoder_{bart}\left(T\right)$$

### 3.3. Knowledge Module

In the Section 3.3, we designed a knowledge prediction approach that integrates reasoning. It aims to predict the knowledge triplets that may appear in the generated responses based on the given context, triplets, relations, and inferred entities. The knowledge module is divided into several steps. Firstly, we utilize the TransD algorithm to represent the entities and relations of the knowledge graph. Then, we predict the relation sequence based on all the relations observed in the context. Subsequently, we perform graph traversal using this relation sequence to obtain the inferred entities for reasoning. Finally, these inferred entities are used to guide the prediction of the knowledge triplets. Next, we provide a detailed explanation.

#### 3.3.1. Knowledge Representation

We use the KG embedding algorithm TransD [16] to represent KG entities and relations. The TransD embedding function provides two mapping matrices that project the head and tail entities. These mapping matrices are jointly determined by entities and relations, making them distinct yet interactive. Compared to TransR, TransD not only considers the diversity of relations but also takes into account the diversity of entities, providing a more comprehensive representation of knowledge.

#### 3.3.2. Relational Sequence Prediction

To capture more information to assist in relation prediction, we utilize a Bidirectional Gated Recurrent Unit (Bi-GRU) [31] to obtain hidden states that are contextually aware of relationships.

We take all the relations that have appeared in the context as input, and the Bi-GRU performs computations separately in both the forward and backward directions. At time step *t*, the final hidden state is obtained by concatenating the output hidden states from the forward and backward directions as follows:

(3) $$h_{r}^{GRU}\left(t\right)=\left[h_{r}^{fw}\left(t\right);h_{r}^{bw}\left(t\right)\right]$$

The formulas for computing the forward and backward hidden states are as follows:

(4) $$h_{r}^{fw}\left(t\right)=\overset{}{GRU}\left(r_{t},h_{r}^{fw}\left(t-1\right)\right)$$

(5) $$h_{r}^{bw}\left(t\right)=\overset{}{GRU}\left(r_{t},h_{r}^{bw}\left(t+1\right)\right)$$

The notation $GRU\left(∙\right)$ represents the *GRU* function. Then, the last layer’s hidden state is used as the input to the relation layer, with the parameter $W_{r}$. The purpose is to predict the relation sequence $R=\left\{r_{h}\right\}(1\leq h\leq H)$, where $r_{h}$ represents the relation used at the *h*-th step during the inference process, and *H* is the maximum number of steps.

(6) $$R=W_{r}^{T}\left(h_{r}^{GRU}\left(t\right)\right)$$

#### 3.3.3. Knowledge Graph Reasoning

We adopt the specific KG representation from [13], which not only reduces memory consumption but also allows the model to scale to larger KGs. Specifically, we use three sparse matrices to represent the knowledge graph KG: the head matrix $M_{h}$, the relation matrix $M_{r}$, and the tail matrix $M_{t}$. Entries with a value of 1 in $M_{h}$ or $M_{t}$ indicate that the *i*-th triple in the KG has the entity *e* as its head or tail, respectively. Entries with a value of 1 in $M_{r}$ indicate that the *i*th triple in the knowledge graph has the relation *r*.

After predicting the relation sequence *r*, we initiate the graph traversal from a set of given initial entities $e_{1}$. We represent the initial entities using a tensor of shape (1, *N_E*), where *N_E* represents the total number of entities. Then, we employ the Reason module to predict the next (temporary) entity vector $e_{2}$:

(7) $$e_{h+1}^{r}=Reason\left(e_{h},r_{h}\right)$$

where

(8) $$Reason\left(e_{h},r\right)=\frac{M_{t}^{T}\left(M_{h}e_{h}⊙M_{r}r_{h}\right)}{||M_{t}^{T}\left(M_{h}e_{h}⊙M_{r}r_{h}\right)|{|}_{2}+\varepsilon }$$

where the symbol ⊙ denotes element-wise matrix multiplication, which refers to multiplying corresponding elements of two matrices to obtain a new matrix. ε represents an arbitrary decimal number. After completing *H*-hop reasoning, entities with the top k values are selected from the entity vector $e_{H}$, which correspond to the entities retrieved with the highest probabilities from the graph. Then, the embeddings of these entities are obtained and multiplied by their values in $e_{H}$. Finally, the embeddings of these entities are combined with the predicted triplets using multi-head attention.

#### 3.3.4. Entity-Aware Triplet Prediction

Similar to relations, given the triplets in the context of *n* − 1 rounds, we calculate the hidden states of each triplet using BI-GRU. The computation process for the *i*th round is as follows:

(9) $$h_{t}^{GRU}\left(i\right)=\left[h_{t}^{fw}\left(i\right);h_{t}^{bw}\left(i\right)\right]$$

(10) $$h_{t}^{fw}\left(i\right)=\overset{}{GRU}\left(t_{i},h_{t}^{fw}\left(i-1\right)\right)$$

(11) $$h_{t}^{bw}\left(i\right)=\overset{}{GRU}\left(t_{i},h_{t}^{bw}\left(i+1\right)\right)$$

After obtaining the predicted hidden states of *n* − 1 triplets and inferred entities, we employ multi-head attention [16] to focus on entity-related triplets. The final triplet obtained is denoted as $h_{t_{n}}^{MHA}$. The computational procedure is as follows:

(12) $$h_{t_{n}}^{MHA}=MultiHead\left(ent_{n},h_{t_{i}}^{GRU},h_{t_{i}}^{GRU}\right)$$

where $MultiHead\left(∙\right)$ denotes the multi-head attention calculation function. Then, we map the predicted triplets to their corresponding labels. We define the predicted label as $l=W_{l}(h_{t_{n}}^{MHA})$, and the true label *y* is a binary vector of dimension $T_{N}$, where each element can take the values of 0 or 1. Here, $T_{N}$ represents the total number of triplets. For computing the loss, we utilize the binary cross-entropy loss function.

(13) $$L_{triplet}=-\frac{1}{T_{N}}\sum_{i=1}^{T_{N}}\left[y_{i}log\left(\sigma \left(l_{i}\right)\right)+\left(1-y_{i}\right)log\left(1-\sigma \left(l_{i}\right)\right)\right]$$

where σ(·) is the sigmoid function, which compresses the input values between 0 and 1, used to represent probabilities. It compares the predicted values and the true values for each element, computes the cross-entropy loss, and ultimately calculates the weighted average of the loss values for all elements to obtain the final loss.

### 3.4. Knowledge-Augmented Decoder

We use the BART decoder as the decoder for our model. Domain knowledge can be injected into the generated responses by combining pre-encoded dialog context $h^{C}$ and related knowledge text $h^{T}$ with predicted knowledge triples $h_{t_{n}}^{MHA}$ and feeding them into the decoder. The formula is as follows:

(14) $$G=Decoder_{bart}\left(\left[h^{C};h^{T};h_{t_{n}}^{MHA}\right]\right)$$

where response *G* represents the final generated response and $Decoder_{bart}\left(∙\right)$ denotes the BART decoder. For the loss function, we employ cross-entropy loss, which is formulated as follows:

(15) $$L_{gen}=-\frac{1}{T}\sum_{t=1}^{T}log\left(P\left(G_{t}=Y_{t}\right)\right)$$

where T means time step, $G_{}$ represents the response predicted by the model, and $Y_{}$ is the label response for reference. The total loss is the sum of triplet predicted loss and response generated loss:

(16) $$L_{total}=L_{triplet}+L_{gen}$$

## 4. Experiments

### 4.1. Dataset

During the experimental phase, we need a dataset consisting of Chinese dialogue and external knowledge, in which each set of conversations should have enough rounds, and each round should be annotated with relevant knowledge. So, we selected the kdconv dataset [10] to validate our model. kdconv is a Chinese knowledge dialogue dataset that consists of 4.5 K dialogues with an average of 19 dialogue turns. It encompasses domains such as music, film, and travel. Each sentence in the dataset is annotated with relevant triplets and knowledge text, establishing a mapping between dialogue and knowledge. Due to the fact that our work does not involve multi-domain issues, we only utilized the multi-turn dialogue dataset from the travel domain. The travel domain knowledge comprises 10 K triplets and 1.1 K knowledge texts.

### 4.2. Baselines and Implementation Details

We compared the KRP-DS model with the following baseline models:

- HRED [18] encodes the entire dialogue history into a context vector and feeds it to the decoder to generate a response.
- HRED + know [10], based on the HRED model, integrates the context vector with knowledge vectors and feeds them to the decoder for response generation.
- BART [30] is a pretrained seq2seq model with powerful performance, being particularly adept at text-generation tasks.
- BART + know [11], built upon the BART model, combines the encoded representation of the context with the average word embeddings of relevant knowledge entities and relations.

We implemented our model using the PyTorch framework. To obtain entity and relation embeddings, we utilized the TransD model provided by OpenKE [32], where the embedding size for entities and relations in the knowledge graph is set to 200. OpenKE is an open-source knowledge embedding framework developed by THUNLP that provides a code implementation of the TransD algorithm. In the knowledge prediction module, we employed a BI-GRU hidden layer of size 300 with one layer, and the maximum number of hops for the relation layer inference was set to 3. For multi-head attention, we used eight attention heads. For the encoder–decoder part, we utilized the Chinese BART model [33], which is implemented by the Hugging Face Transformers library, with default hyperparameter settings. During the decoding process, we employed beam search with a beam size of 5. The maximum length of the generated text was set to 150, and the batch size was set to 8. We used the Adam optimizer with an initial learning rate of 5 × 10^−5^.

### 4.3. Evaluation Metrics

To comprehensively evaluate the performance of our proposed model, we employed a combination of various automatic evaluation metrics and human evaluation metrics. These metrics not only provide objective quantitative assessments but also offer intuitive insights and in-depth analysis of the response quality.

Regarding the automatic evaluation, we follow the approach of previous work [10] and utilize the following widely used metrics to measure the quality of the generated responses:

- **PPL (Perplexity):** used to measure the predictive power of a language model and the fluency of the generated text. A lower PPL value indicates that the model’s predictions are closer to the real data and the model performs better.
- **BLEU1/2/3/4:** BLEU is a metric used to automatically evaluate machine translation results. It evaluates the quality of translation by comparing the degree of lexical overlap between the generated translation result and the reference translation. BLEU1 indicates the percentage of exact matches for a single word, BLEU2 indicates the percentage of exact matches for two consecutive words, and so on.
- **Distinct-1/2/3/4:** Used to assess the diversity and uniqueness of the generated text. Distinct-1 indicates the proportion of different words in the generated text, Distinct-2 indicates the proportion of two different consecutive words in the generated text, and so on. Higher Distinct values indicate that the generated text is more diverse and unique.

In terms of human evaluation, we followed [10] and conducted evaluations from two aspects: fluency and coherence. Fluency evaluation assesses the naturalness of the generated responses, while coherence assesses whether the generated responses are relevant to the context and consistent with reference knowledge information. We randomly sampled 100 contexts from the test set along with the responses generated by each model, and these were then provided to four well-educated annotators for assessment. For each model, each evaluator assigned scores to the 100 responses generated by that model based on the evaluation criteria described above for the two aspects mentioned. The average score was then computed. The final score for each model was obtained by averaging the scores given by the four evaluators. The specific evaluation criteria are presented in Table 2.

### 4.4. Results and Analysis

Table 3 presents the automatic evaluation results of all models on the kdconv dataset. Our model outperforms the baseline models in most metrics, demonstrating its ability to generate more diverse and higher-quality responses.

In the majority of metrics, the performance of BART-related models is superior to that of HRED-related models, indicating that pre-trained models possess stronger conversational abilities compared to traditional models. Furthermore, all models that incorporate additional knowledge exhibit higher BLEU and Distinct scores compared to models without external knowledge. This indicates the effectiveness of external knowledge, as augmenting models with external knowledge can facilitate the generation of higher-quality responses. Our model outperforms the BART + Know model on all metrics except for PPL and Distinct-1, indicating the effectiveness of our knowledge module.

Our model performs worse than the BART-related models in terms of PPL. This may be attributed to the fact that we consider more knowledge, resulting in more diverse generated responses that are less common in the dataset. Consequently, the model struggles to comprehend them, leading to lower PPL scores.

Our model outperforms the BART-related models in terms of the Distinct-1 metric but falls behind the HRED-related models. This could be attributed to the fact that HRED-like models tend to group together irrelevant low-frequency words, as the higher the value of Distinct-1, the more completely random words are brought together.

Due to the unique nature of dialogues, the evaluation metrics for automatic assessment can only examine the model’s performance from certain aspects, which has certain limitations. Therefore, we will further analyze the results based on human evaluations.

Table 4 shows the results of the human evaluation, with our model receiving the highest scores for both fluency and relevance. The HRED + Know model has fluency and relevance scores of 1.63 and 1.25, respectively, indicating that the model generates responses that are relatively fluent, but are often irrelevant to the context and use knowledge that is inconsistent with real knowledge information. The BART + Know model has fluency and relevance scores of 1.81 and 1.52, indicating that the model has been able to generate fluent responses. However, the problems of responses not being relevant to the context and utilizing incorrect knowledge still occur from time to time. On the other hand, the fluency and relevance scores of our KRP-DS model are 1.92 and 1.75, indicating that the model generates fluent responses that are contextually relevant in most cases, and that the knowledge information used is reasonable and consistent.

Finally, we computed the Fleiss’ kappa value for Fluency and Coherence. This value is used to measure inter-assessor agreement, and ranges from [−1, 1]. Higher values indicate better inter-assessor agreement. We calculated the results as 0.72 and 0.76, respectively, indicating that a good inter-assessor agreement was achieved.

## 5. Case Study

We take BART + Know and KRP-DS models as representatives and select the two-round responses generated by them as examples. In the first round, A’s response is generated, and then the label response of A is added to the context for the generation of B’s response in the second round. The examples of the responses are shown in Table 5.

It is easy to observe that the responses generated by BART + Know are contextually relevant but prone to incorporating erroneous knowledge. Additionally, these responses exhibit poorer coherence with the preceding text and lack smooth transitions between dialogues. On the other hand, the responses generated by our KRP-DS model are more natural and fluent, while are also accurately selecting appropriate knowledge. Our KRP-DS model utilizes a knowledge module to predict appropriate knowledge, which is then used to enhance response generation, ultimately improving the quality of the responses. Furthermore, our model is capable of extracting interpretable knowledge reasoning paths, thereby enhancing explainability.

## 6. Error Analysis

In order to have a deeper understanding of our model, we collected some incorrect responses generated by KRP-DS as in Table 6, performed an error analysis, and further explore how to make improvements in the future.

We found that the vast majority of failed responses are applying the wrong knowledge. Especially when the external knowledge involves numerical correlations, such as tickets, opening hours, phone numbers, etc., the model often chooses to predict the wrong knowledge and generates wrong responses. Our analysis reveals that this can be caused by a number of factors. First, for numbers and similar entities, the TransD algorithm may have limited representation capabilities, leading to inaccurate representations that prevent the model from distinguishing between them. Second, insufficient training data may be an issue. There may be fewer or less diverse examples involving such entities in the training data, resulting in the model’s inability to learn the representation correctly and generate accurate responses. Finally, the decoder part of the model is also a key factor. Currently, our approach feeds correct knowledge into the decoder and waits for a response to be generated, but we cannot be sure that the model will necessarily utilize this knowledge correctly. This is because the decoder part of BART uses an autoregressive mechanism in the generation process, word by word, and has no direct control over the specific knowledge input.

Based on these, we found directions for improvement for future work. The first one is to optimize the external knowledge representation, improve the accuracy and quality of the external knowledge represented by transd, or find a new knowledge representation. The second is to refine the shortcomings of this dataset, add what is missing, and build a larger and more diverse knowledge dialogue dataset based on it. The third is to add a knowledge validation module after the decoder to verify that the knowledge in the generated responses is correct and to fix the wrong responses.

## 7. Conclusions

In recent years, with the continuous development of pre-training techniques, research on dialogue systems have made significant progress. However, current models often lack domain-specific knowledge and interpretability. In this paper, we propose a new knowledge-based dialogue system model called KRP-DS. We design a knowledge module that combines reasoning capabilities with prediction, thus achieving both predictive ability and improved interpretability. In the decoding and generation phase, we use predicted knowledge to guide response generation, thereby enhancing the quality of the responses. Experimental results demonstrate that our model generates high-quality responses with interpretable KG reasoning paths, leveraging more relevant knowledge and making them more appealing. In future work, we will explore ways to maintain response richness while reducing hallucinations and improving response authenticity.

## Figures and Tables

**Table 1:** Ordinary responses and responses enhanced with external knowledge.

Conversation	
Context	I like Nanluoguxiang very much, it is one of the fashion landmarks in Beijing.
Response	BART: I really don’t know about this.
	KRP-DS (Ours): Well, so young people, especially those who are into arts and literature, often consider this place a must-visit when exploring Beijing.
Context	How long does one typically spend there when visiting?
Response	BART: I really don’t know about this.
	KRP-DS (Ours): 1 h–2 h.

**Table 2:** Human evaluation standards.

	Score	Standards
**Fluency**	0	Serious grammatical errors, not fluent and difficult to understand
	1	There are some grammatical errors, but they are understandable
	2	It’s smooth, as though it is created by humans
**Coherence**	0	Context independent
	1	Contextually relevant, but inconsistent with knowledge information
	2	Contextually relevant yet consistent with knowledge information.

**Table 3:** Automatic evaluation. The optimal result for each indicator is highlighted in bold.

Model	PPL	Bleu-1	Bleu-2	Bleu-3	Bleu-4	Distict-1	Distict-2	Distict-3	Distict-4
HRED	10.90	30.92	20.97	15.61	12.30	**4.15**	12.01	20.52	28.74
BART	1.83	34.77	29.11	25.69	23.33	2.70	13.39	21.92	29.53
HRED + know	11.15	36.87	26.68	21.31	17.96	3.98	13.31	24.06	34.35
BART + know	**1.67**	36.19	29.83	26.04	23.41	2.59	13.31	22.01	29.69
KRP-DS	2.08	**45.00**	**38.52**	**34.69**	**32.09**	2.71	**15.82**	**26.59**	**35.31**

**Table 4:** Human evaluation result.

Model	Fluency	Coherence
HRED + Know	1.63	1.25
BART + Know	1.81	1.52
KRP-DS	1.92	1.75

**Table 5:** Conversation examples.

Context	Knowledge
**A:** Nanluoguxiang, as one of Beijing’s fashionable landmarks, is definitely worth a visit. **B:** Moreover, it is a hutong that combines a bourgeois atmosphere with the old Beijing charm. **A:** Moreover, the duration of 1 to 2 h for sightseeing is just right, as it won’t make people feel tired. **B:** Sure, after visiting here, you can proceed to explore the adjacent Prince Gong’s Mansion. **A:** Prince Gong’s Mansion, as the largest royal mansion of the Qing Dynasty, is truly worth a visit.	**Knowledge triplets:** (Nanluoguxiang, Recommended play time, 1 h–2 h.) (Nanluoguxiang, Attractions nearby, Prince Gong’s Mansion) **Knowledge text:** Nanluoguxiang is located in the center of Beijing…… Prince Gong’s Mansion is located on Qianhai West Street, Xicheng District, Beijing. It is the largest royal mansion of the Qing Dynasty……
**B label response:** How much is the admission ticket again?	
**BART + know:** How long is the recommended duration for visiting it?	
**KRP-DS:** Yes, it is a must-see. Do they charge an admission ticket fee here?	
**A label response:** 40 yuan.	
**BART + know:** High season 60 yuan, Low season 40 yuan.	
**KRP-DS:** 40 yuan.	**Knowledge triplets:** (Prince Gong’s Mansion, Admission ticket, 40 yuan) **Reasoning Path:** $Nanluoguxiang\overset{Attractions\;nearby}{\to }Prince\;Gong\prime s\;Mansion\overset{Admission\;ticket}{\to }40\;\mathrm{yuan}$

**Table 6:** Example of error response generated by the model. The italicized underline indicates the error knowledge entity used in the response.

Context	Response
Hello, have you been to the Poly Art Museum? … Do you know the phone number?	**Label response:** 010-65008117. **KRP-DS:** The phone number is *010-59789114*.
The King Gong’s Palace was the largest of its kind in the Qing Dynasty… When exactly was it opened?	**Label response:** High season, 1 April–31 October, 8:00–17:00; Low season, 1 November–31 March, 9:00–16:00. **KRP-DS:** *8:00–16:00*.
Do you know anything about Yuetan Park? … What other attractions are there around it?	**Label response:** The Imperial Palace, ah, the most worthwhile attraction. **KRP-DS:** The *National Museum of China* is well worth a visit.

## Data Availability

Not applicable.
